# Analysis of the Mechanical Properties of the Human Tympanic Membrane and Its Influence on the Dynamic Behaviour of the Human Hearing System

**DOI:** 10.1155/2018/1736957

**Published:** 2018-05-09

**Authors:** L. Caminos, J. Garcia-Manrique, A. Lima-Rodriguez, A. Gonzalez-Herrera

**Affiliations:** ^1^Departamento de Ingeniería Mecánica, Universidad Nacional Experimental del Táchira, San Cristobal, Venezuela; ^2^Departamento de Ingeniería Civil, de Materiales y Fabricación, Universidad de Málaga, Malaga, Spain

## Abstract

The difficulty to estimate the mechanical properties of the tympanic membrane (TM) is a limitation to understand the sound transmission mechanism. In this paper, based on finite element calculations, the sensitivity of the human hearing system to these properties is evaluated. The parameters that define the bending stiffness properties of the membrane have been studied, specifically two key parameters: Young's modulus of the tympanic membrane and the thickness of the eardrum. Additionally, it has been completed with the evaluation of the presence of an initial prestrain inside the TM. Modal analysis is used to study the qualitative characteristics of the TM comparing with vibration patterns obtained by holography. Higher-order modes are shown as a tool to identify these properties. The results show that different combinations of elastic properties and prestrain provide similar responses. The presence of prestrain at the membrane adds more uncertainty, and it is pointed out as a source for the lack of agreement of some previous TM elastic modulus estimations.

## 1. Introduction

The function of the tympanic membrane (TM) in the sound transmission process is easy to understand intuitively. The piston-like motion, which transfers air sound pressure wave into the cochlea, has been clearly identified long time ago [1, 2]. Nevertheless, at higher frequencies, the TM motion is not so simple and new characteristic patterns appear.

There have been many experimental and numerical studies to evaluate the behaviour of the TM. These patterns have been widely studied experimentally since the development of techniques as holography [3–5]. With this technique, the complex TM vibration patterns at high frequencies have been revealed.

The alternative is numerical simulation. Since the early works led by Funnell and Laszlo [6] and Funnell et al. [7, 8], finite element (FE) models have been used to study the behaviour of the system [9–19]. Nevertheless, uncertainties regarding the accuracy of the material properties hinder the numerical simulation and limit the extension of the conclusions. This is particularly significant in the case of the TM.

A great deal of work has been devoted to estimate these properties, especially to the determination of the TM elastic modulus (EM). Most of the results range from 20 to 40 MPa or are close to these values. They are based on tension tests on small samples [1, 2, 20, 21] and are subject to important uncertainties. Different indentation techniques have been developed providing result in the same range [22–25]. Nevertheless, recently, values on the range of 3 MPa have been reported [26], showing the difficulty to obtain accurately this parameter separately from the estimation of the thickness.

Fay et al. [27] suggested that these values could be underestimated; they used composite laminate theory and evaluated their result comparing with the dynamic response of the TM. They calculated the effect of the orientation of the fiber on the EM obtained on tension or bending tests when an isotropic assumption was made. They showed that the EM could be in a broad range depending of the angle of orientation of the fibers of the sample tested. They confirmed their finding by means of correlating experimental dynamic wavelength pattern. These patterns were obtained from live anesthetized cat but conclusions can be transferred to the human case. Fitting a mathematical model and by means of a parameter estimation procedure, they suggested that the EM should be in the range 0.1 to 0.3 GPa.

A potential reason for this high stiffness value could be the presence of an unaccounted prestrain. It is well accepted the presence of active muscles on the middle ear system [28], but apart from hypothesized active tension effects inside the TM due to smooth muscles [29], only passive prestrain has been named by several authors [1, 2], but no quantitative estimation has been clearly made. Recently, Aernouts and Dirckx [30] assumed an in situ strain for the elastic characterization of the gerbil pars flaccida, but no other numerical model has included this effect.

In the present paper, the parameters that define the bending stiffness properties of the membrane have been studied. Mainly, the influence of two parameters has been studied: the Young's modulus of the tympanic membrane and the thickness of the eardrum. Additionally, it has been completed with the evaluation of the presence of an initial prestrain inside the TM.

Firstly, a harmonic analysis is made to evaluate the range of influence of these properties. Secondly, a modal analysis is used to check how the properties affect the dynamic response. We will demonstrate that variations in membrane parameters have small effects on the lower natural frequencies; the effects of thickness, EM, and prestrain on the higher natural frequencies are larger and separable.

Rosowski et al. [4] used holography to observe the TM vibration pattern in different species including human. They established a qualitative description based on the pattern observed and related with the frequency range. They considered a simple pattern when only one maximum displacement was observed (below 2 kHz in human), complex pattern when more than one maximum (from 2 to 8 kHz), and ordered pattern when a high number of maximum appear (above 8 kHz). This description will be used to evaluate the response of the TM at different ranges of frequencies.

Direct comparison of numerical and experimental results is not easy to do [31, 32] as the acoustomechanic coupling must be solved; nevertheless, comparing numerical modes and experimental vibration pattern leads to identify which of these material properties combinations are more suitable and suggest potential future working lines.

## 2. Materials and Methods

### 2.1. FEA Modelling Methodology

A brief summary of the main modelling methodology is presented in this section (see detail in [16]). It has been developed from precedent works [6–15].

Two models will be described. The first one includes all the elements of the middle ear, referred as tympanic-ossicular system (TOS) model. The second one only includes the TM and the approximate effect of the manubrium. In both cases, linear elastic material behaviour and small displacement condition are assumed. Both models have been simplified and limited to the components necessary for the purpose of this study.

**Figure 1:**
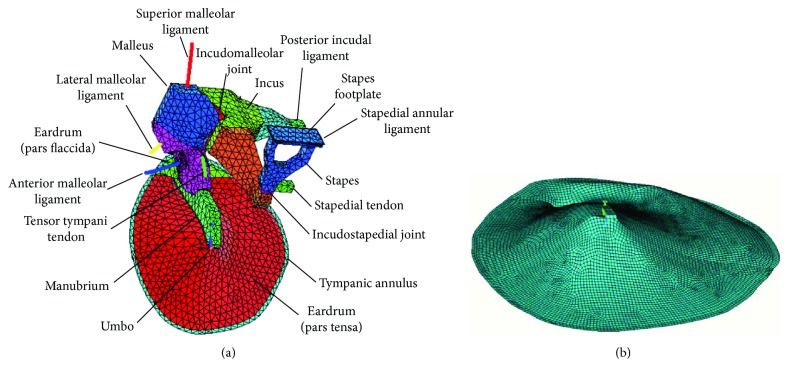
Finite element models: (a) tympanic-ossicular system (TOS) model and (b) tympanic membrane simplified model.

The anatomic measures and functional properties were based on published data. The geometrical model is divided into three parts: TM, ossicular chain, and the system of ligaments, tendons, and joints (Figure 1(a)). Details of the geometry and property estimation are described in different references [16, 33].

The FE model was developed using ANSYS. TM was modelled with shell elements assuming uniform thickness (50 *μ*m) in order to simplify the analysis. The tympanic annulus is modelled as a band (0.2 mm wide and 0.2 mm thickness) with the external border fixed. Solid elements are used for the ossicular chain, posterior incudal ligament, and incudomalleolar and incudostapedial joints. Ligaments and tendons supporting malleus are modelled with beam elements. The stapedial annular ligament is modelled with shell elements surrounding the stapedial footplate (0.05 mm wide and 0.05 mm thickness). Figure 1(a) shows the middle ear FE model, and the mechanical properties are summarized in Table 1.

The effect of the impedance of the fluid of the cochlea was modelled using a mechanical equivalent load consisting of a block of mass and dashpots according to [14]. This simplified model provides a good accuracy with a low computational cost. When modal analysis is applied to the TOS model, this equivalent load is removed to eliminate its own vibration modes, which are outside the scope of this study.

Regarding the TM simplified model (Figure 1(b)), most of the description previously stated is valid. The area corresponding to the connection with the manubrium has also been meshed with shell elements, but the mechanical properties have been adapted to represent the inertial and stiffness effect of the ossicular chain. The umbo was represented by an area 0.6 mm wide with an EM equal to 14 GPa and the density 1900 kg/m^3^. In this way, the ossicular chain complex vibration patterns—out of the scope of this part of the study—are excluded from the model. Another important difference is that the membrane has been meshed with a higher number of elements as this model is intended to capture accurately high-frequency vibration pattern.

### 2.2. Middle Ear Harmonic Response

As a starting point of this study, a sensitivity analysis has been done in order to evaluate the influence of two key mechanical properties that strongly influence the response of the system: TM elastic modulus and TM thickness.

**Figure 2:**
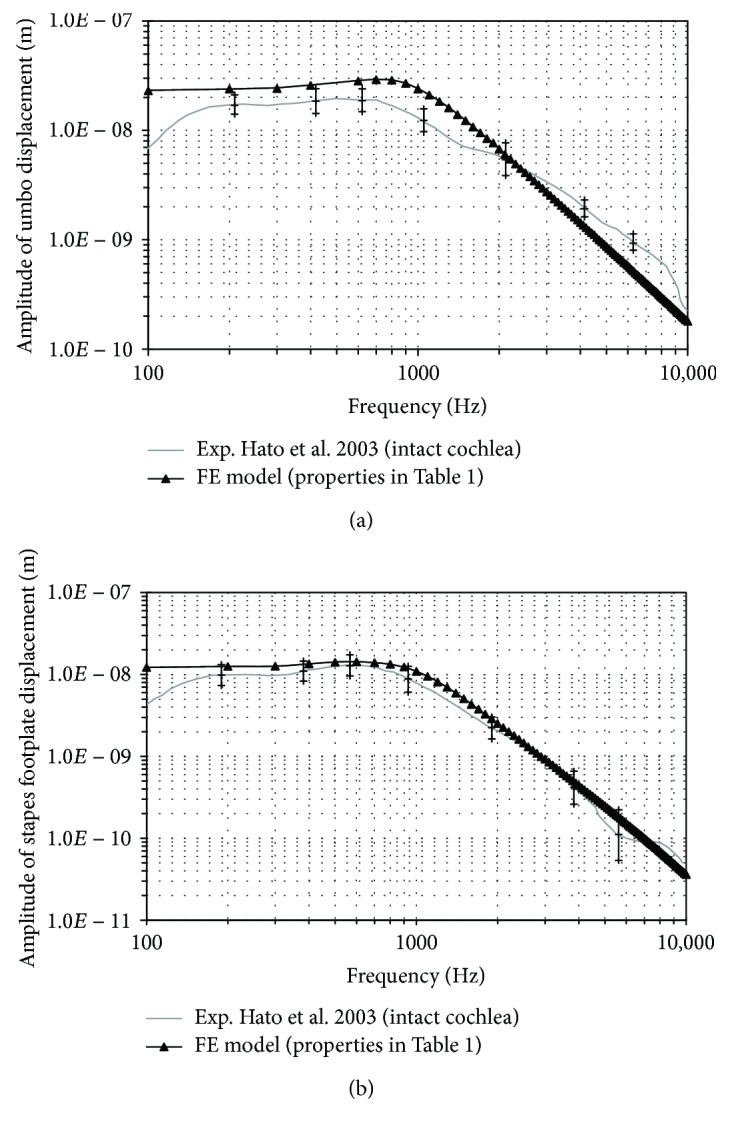
Amplitude of displacement versus frequency of umbo (a) and stapes footplate (b), for a range from 100 to 10,000 Hz at 80 dB_SPL_ sound pressure.

First, the behaviour of the middle ear model will be shown. A harmonic analysis was conducted from 100 to 10,000 Hz. A uniform harmonic 80 dB_SPL_ stimulus pressure was applied to the lateral side of the eardrum. The amplitude of umbo and stapedial footplate displacements versus frequency is shown in Figure 2 compared with experimental measurements [37]. These experimental curves are the average data of 10 human temporal bones with intact cochlea, using LDV with 80 dB sound pressure applied at the TM in similar condition to the present study. Rayleigh damping *β* = 0.0001 s is assumed. With Table 1 parameters, the numerical result approximately predicts the experimental displacement.

**Figure 3:**
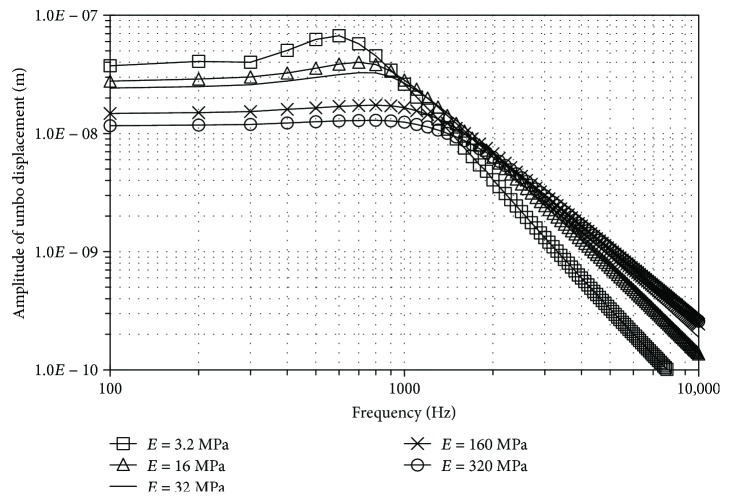
Influence of the TM elastic modulus of the pars tensa at umbo, for a range from 100 to 10,000 Hz at 80 dB_SPL_ sound pressure.

**Figure 4:**
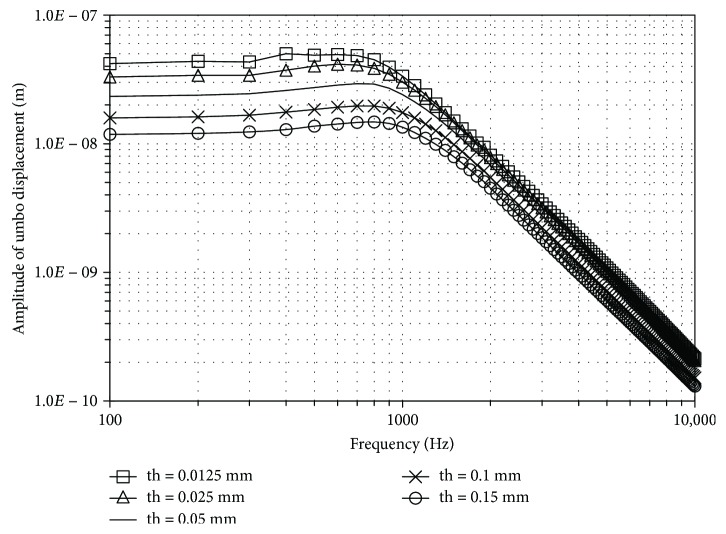
Influence of the thickness of tympanic membrane at umbo, for a range from 100 to 10,000 Hz at 80 dB_SPL_ sound pressure.

Figures 3 and 4 show the influence of the EM and the thickness, respectively. The thick line without marks corresponds to the results obtained with the reference model (Figure 2, Table 1). Values assigned to EM range from 3.2 MPa to 320 MPa, covering the extreme values obtained from literature.

The TM EM presents a qualitatively similar influence in both cases (Figure 3). Below 1000 Hz, the displacements increased as the stiffness decreases. The peak value is at a lower frequency with a lesser stiffness. The displacement at high frequency (above 2000 Hz) presents an opposite relation; it increases slightly as the stiffness increases.

Regarding the thickness, its response is inversely proportional to the increase of stiffness and mass due to the increment of thickness (Figure 4). The influence is more significant at lower frequencies. The peak value close to 1000 Hz is similar for all curves.

These results are similar to those obtained by Gan et al. [14] suggesting that an isotropic material behaviour as well as a mean thickness for the tympanic membrane model can fit experimental data for the frequency range studied.

According to the results shown, it could be stated than 32 MPa seems to be a good value for the TM EM; nevertheless, important variations of this parameter lead to result that could be argued as valid too, depending of the experimental data used to compare.

## 3. Results

**Figure 5:**
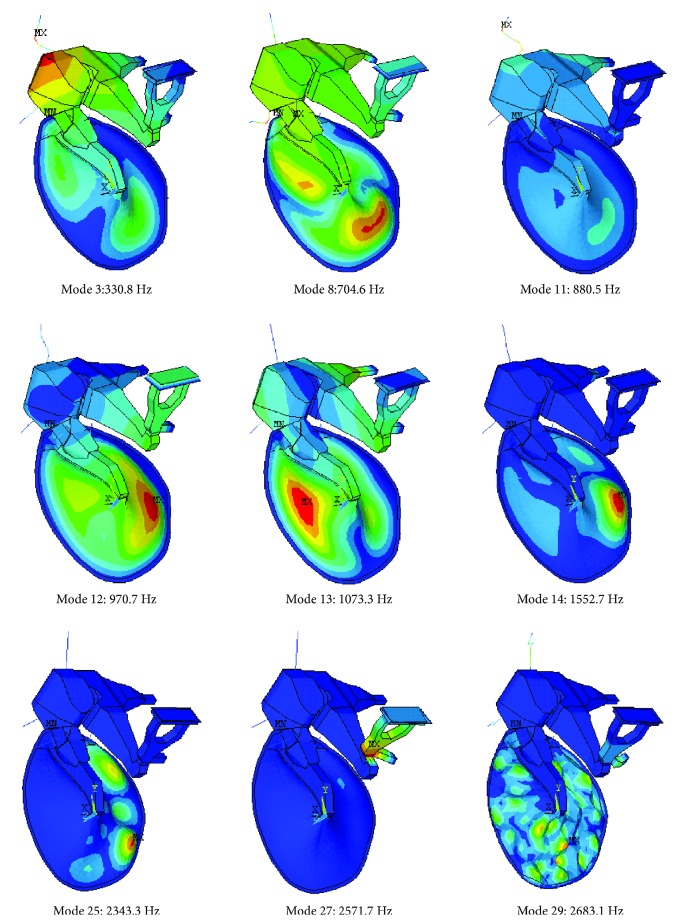
Selected mode shapes. Middle ear FE model including the tympanic-ossicular system (TOS). TM elastic modulus *E* = 32 MPa; thickness th = 50 *μ*m.

Different modal analyses were performed. As a reference, a modal analysis of the whole system (TOS) was made. Some selected mode shapes are drawn in Figure 5. Different types of shapes are present. Some clearly reflect a TM vibration pattern (modes 8, 12, 13, 14, 25, and 29). Mode 12 is the classic piston-like motion. Mode 8 presents a similar membrane motion but with a lower stapes displacement. Modes 3 and 11 show the movement of the joints and ossicles while the membrane and the stapes present a very low motion. Mode 27 shows a tilting movement of the stapes with hardly no membrane displacement. Modes 14 and 25 correspond to the transition to *complex* pattern (as described in Rosowski et al. [4]), and mode 29 would be the starting point of the *ordered* pattern. In this model, high-frequency modes are less accurate than lower due to the limited number of element used. Nevertheless, they are a good reference as it was proved with TM models.

A list of the 30 first modal frequencies is in Table 2, where the type of dominant pattern has been marked (due to TM; joint, J; or ligament and tendon motion, LT). This classification is made based on the part of the system where higher motion is present. Different mechanisms will contribute to the sound transmission in this frequency range. The sound pressure stimuli at the membrane will make dominant those modes involving the TM motion. The presence of the other modes will be lower or negligible.

The TM simplified model was developed in order to have the opportunity to make many calculations with a reasonably computational cost and enough accuracy at higher frequencies. It also provided the chance to compare with previously published results (see [38]). The modal analysis was performed with this model. As the ossicular chain has been removed, only the TM modes are present on the solution.

**Figure 6:**
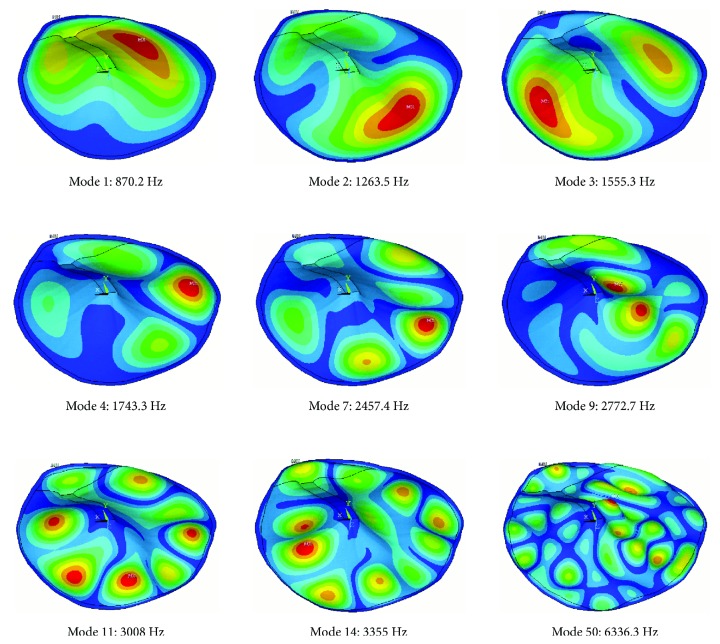
Selected mode shapes. Tympanic membrane FE model. TM elastic modulus *E* = 32 MPa; thickness th = 50 *μ*m.

Figure 6 presents some selected mode shapes calculated with the reference properties (Table 1). It can be seen how modes 1, 2, and 3 (Figure 6) are equivalent to modes 12, 8, and 13, respectively (Figure 5) and can be considered *simple* patterns. Mode 14 (Figure 5) is equivalent to mode 4 (Figure 6) and mode 5 (not drawn), being the first *complex* patterns. Modes 11 and 14 (Figure 5) are representative of the starting point of the transition zone to *ordered* pattern. Finally, mode 50 corresponds clearly to the *ordered* pattern, where a huge number of vibration modes can be observed with similar shapes. The small element size used in this model to mesh the TM captures these modes very accurately. At this frequency range, the absence of the ossicular chain has a reduced influence [18, 19], so for the purpose of the present study, this simplified model is acceptable. As a rule of thumb for the interpretation of the following figures, mode 5 may represent the transition to *complex* pattern and mode 15 the transition to *ordered* pattern.

**Figure 7:**
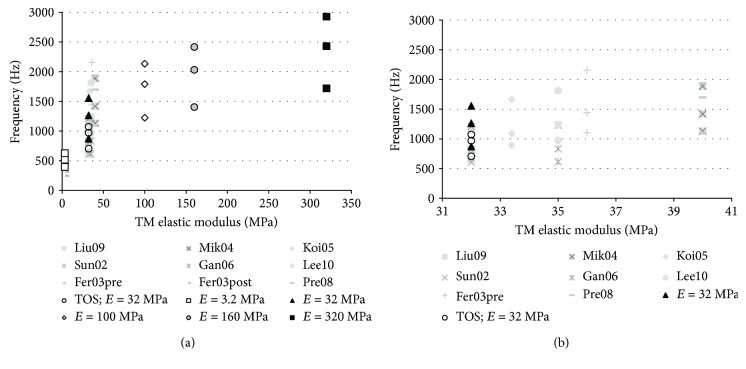
(a) 1st, 2nd, and 3rd tympanic membrane modal frequencies in terms of the TM elastic modulus and (b) scale enlarged.

In order to clarify the influence of the TM elastic modulus on the dynamic response, modes 1, 2, and 3 have been plotted in Figure 7. Results provided by Volandri et al. [38] have also been included.

The same references have been used (see [38] to consult the original references). The main idea we can extract from these figures is something somehow expected. Considering some exceptions due to particular modelling circumstances (as the use of different TM thickness or boundary condition), we can see how the first frequencies are related to the stiffness of the membrane.

Since each model provides different results, it is difficult to argue which of them is the best. Excepting at very low elastic modulus, which can be discarded as unreal, the other results could be considered acceptable as they are ranging on the different proposed elastic modulus. This is the same conclusion obtained from the sensitivity analysis on previous section.

**Figure 8:**
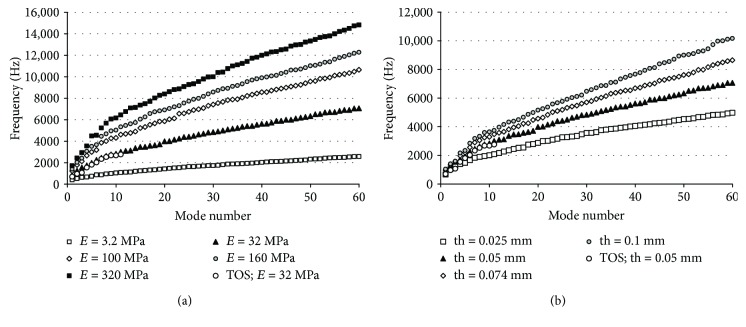
Tympanic membrane modal frequencies in terms of the (a) TM elastic modulus and the (b) TM thickness.

Nevertheless, if we observe the behaviour of higher modes, we can make some distinction between these results comparing with the experimental observation. In Figure 8, the values of the first 60 TM vibration modes are plotted and calculated with different elastic moduli (Figure 8(a)) and different TM thickness (Figure 8(b)). If we observe the curve corresponding to the reference values (*E* = 32 MPa, black triangle), the first thing we can see is how the whole system results (TOS) are coincident with the simplified model.

However, if we focus on the higher modes, now we can detect significant differences with the experimental results observed. Considering mode 50 (Figure 8) as a reference, this type of pattern has been detected in human at frequencies above 8 kHz (see Figure 4 in [4]); in this case, the frequency 6.3 kHz could be considered very low and wrong. Increasing the value of the elastic modulus (keeping constant the other parameter of the model), we can reach the experimentally observed frequencies. Result obtained in the range 100 to 320 MPa would fit this requirement (Figure 8(a)). This is coherent with the values proposed by [27] for the elastic modulus.

Regarding the influence of the TM thickness (Figure 8(b)), we can see how it hardly affects the lower frequencies but it increases the higher modes. Its effect is not as significant as the elastic modulus, and the range of uncertainty is smaller.

In any case, we can conclude that multiple combinations of both values could lead to similar results as many authors have pointed out. This is the main critique to FE models.

Finally, this TM model has been used to evaluate the influence of a potential prestrain in the membrane. It is another aspect of the problem that has not been considered previously in numerical simulations but presents a significant influence on the results. In this case, it has been considered in the simplest possible way. A homogeneous and uniform isotropic strain value has been applied to the membrane, ranging from 0.1% to 1% (*ɛ*_11_ = *ɛ*_22_ = 0.001 to 0.01 in the plane of the TM and *ɛ*_33_ = − 2 *ɛ*_11_). These values are very below those assumed by Aernouts and Dirckx [30] and cause a static imperceptible displacement of the umbo very below 10 *μ*m. The presence of prestrain must be related to the position and orientation of the fiber on the membrane. A detailed and accurate estimation of a complex prestrain pattern is difficult to do without speculations, so this approach is considered a good solution to evaluate its qualitative influence.

**Figure 9:**
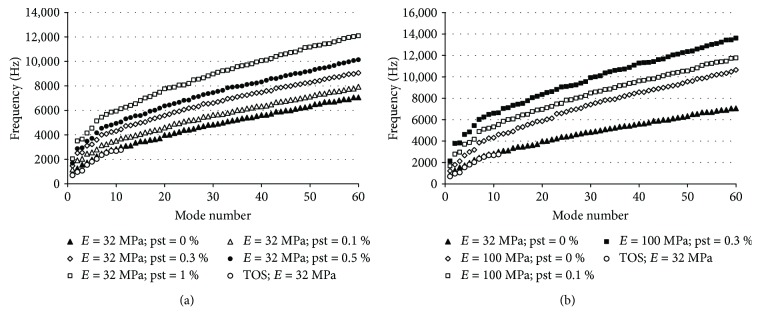
Tympanic membrane modal frequencies with different prestrain (pst) level: (a) TM elastic modulus 32 MPa and (b) TM elastic modulus 100 MPa.

**Figure 10:**
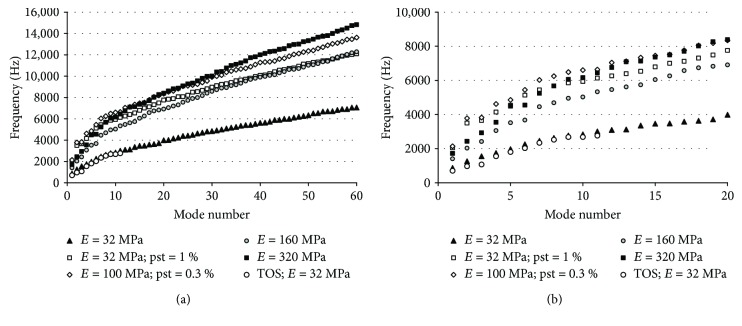
Tympanic membrane modal frequencies with different TM elastic modulus and prestrain (pst) level combination.

Figure 9 shows the result obtained using two different elastic moduli. It can be seen how in both cases the effect is similar to the increase of the elastic modulus. Therefore, again, it is possible to find different combinations of elastic modulus and prestrain that provide similar responses. This has been plotted in Figure 10, where it can be observed how the cases *E* = 32 MPa, pst = 1%; *E* = 100 MPa, pst = 0.3%; and *E* = 160 MPa and *E* = 320 MPa without prestrain provide results in a narrow band which could be accepted as realistic.

It is interesting to observe how the effect of prestrain and elastic modulus increase is different at higher frequencies and at lower (Figure 10(b)). At higher frequencies, the slope of the curve is lower when the increase of the stiffness is due to the prestrain instead of the EM. This different behaviour opens a possible future working line where joint numerical and experimental works could be done in order to fit more accurately the vibration patterns in order to estimate these parameters.

## 4. Discussion

In previous section, a great deal of numerical results has been shown. From them, the main general conclusion is the dependency of the results with the values of the mechanical properties used on the model. This dependency cannot be distinguished due to the general good agreement between numerical and experimental results for a wide range of values. This comparison is normally made in terms of umbo and stapes movement whose experimental measurement is based on the very common and extended LDV technique. No significant differences are observed at lower frequencies, and differences at higher frequencies can be attributable to several modelling issues.

Nevertheless, this lack of accuracy on the estimation of the mechanical parameter has been widely criticized from the side of the experimental researcher and is the main reason to the limited confidence of the results based on numerical models of the hearing system.

In this section, a brief discussion on the consequences of this source of uncertainty on the model and the potential research lines to solve it is made.

According to the results shown previously and regarding numerical modelling, the use of bending stiffness parameters estimation (thickness and elastic modulus) may be acceptable in order to represent the TM behaviour in terms of umbo or stapes displacement, especially at lower frequencies.

It also seems that rather than estimating separately properties very accurately, it would be valid fitting a combination of both properties to describe the bending stiffness of the TM over the whole range of frequency of interest.

**Figure 11:**
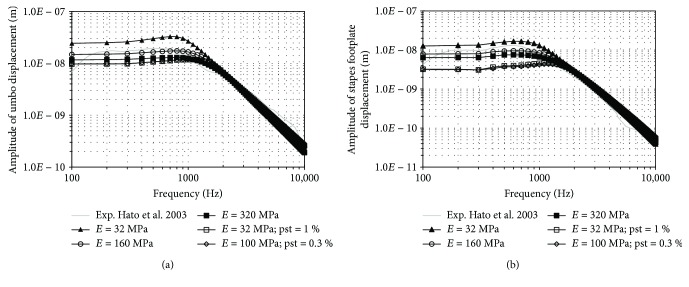
Influence of the prestrain of the TM (a) at umbo and (b) stapes footplate, for a range from 100 to 10,000 Hz at 80 dB_SPL_ sound pressure.

This picture increases its complexity if we include an additional potential effect of stiffness on the membrane: prestrain. The presence of prestrain alters significantly the whole response of the system. This presence is difficult to distinguish from the estimation of the other mechanical properties.  Figure 11 shows the result of a harmonic analysis made with two combinations of parameter *E* = 32 MPa, pst = 1% and *E* = 100 MPa, pst = 0.3%, showing a similar behaviour for the case with higher TM elastic modulus (*E* = 160 MPa and *E* = 320 MPa without prestrain). Even when the reference model (with the properties shown in Table 1) also fits properly experimental data (Figure 2), we should go back to the observation of higher-order modes (Figures 8–10, see Discussion on previous section) to conclude that these new combinations of parameters (including prestrain) are closer to the dynamic behaviour of the membrane (as described by the experiment of [4]).

Experimental work should be done to evaluate and estimate potential prestrain in the TM. Its probable influence on the dynamic response of the TM and the ME has been stated in qualitative terms. It also has been shown as a possible source of discrepancy between statically and dynamically estimation of the elastic modulus.

Elucidating the real presence of this effect is a key aspect to be studied in future as it can help to understand the consequence of some surgical intervention (as tympanoplasty [39]). Different combinations of prestrain in the two TM fiber layers make difficult to speculate about this phenomenon. Detailed numerical models, including laminate theory, should be developed to account for this effect.

Numerical work supported on holography or in LDV measurement along a line should be an option. The high number of combined vibration modes in the range of frequency of interest is a difficulty, but techniques used in dynamic system identification could be adapted for this purpose. It is the case of modal experimental techniques used for model update.

In this sense, proper numerical-experimental techniques should be developed being a key aspect its capacity to account for the acoustomechanic coupling [31]. Experimental displacement curves as shown in Figure 2 [37] are not frequency response function of the mechanical system, because input function on the mechanical system is not measured. They are normalized by the sound pressure at the source, but certain acoustic coupling effects are not included.

Some recent numerical models include this acoustic effect ([15, 17–19]), but even in this case, the condition in which the experiment was made may show significant differences, making the comparison limited. Different conditions on the experiment as the position of the source of sound [31, 32] or the presence of cavities open or closed lead to different responses of the same mechanical system.

## 5. Conclusions

A contribution to evaluate the quality of the parameter estimation of the mechanical properties of the TM on the hearing system has been done in this paper. This is a key aspect when developing numerical models.

Supported on experimental observation provided with holography techniques, especially at high-frequency pattern, TM stiffness properties suggested on bibliography have been evaluated by means of FE numerical models.

One of the conclusion of this work is that different parameter combinations (TM thickness and EM) may lead to similar result that could be considered correct when comparing with experimental data. This makes necessary additional external means of validation to clarify which of these parameters are acceptable.

It also has been shown that prestrain presence causes an increase on the TM stiffness that makes difficult to distinguish its effect from the effect of the elastic modulus of the material. This could be the reason for the lack of agreement of some TM elastic modulus estimation. The different pattern observed due to the prestrain effect would allow distinguishing it from the EM. Adapted modal experimental techniques can be applied to determine this prestrain.

Prestrain effect must be studied and evaluated because even a small value alters significantly the response of the system. Additionally, orthotropic behaviour must be included and probably related to this prestrain.

Numerical and experimental joint works should be done to identify the mechanical properties. In this sense, numerical models must include the acoustic part of the system in order to account for the acoustomechanic coupling effects and to allow numerical-experimental fitting.

## Figures and Tables

**Table 1:** Mechanical properties used in middle ear components for finite element model.

Component	Density (kg/m^3^)	Young's modulus (N/m^2^)	Poisson's ratio
Eardrum			
Pars tensa	1.2 × 10^3^ [9]	3.2 × 10^7^ [10]	0.3 [13]
Pars flaccida	1.2 × 10^3^ [9]	1 × 10^7^ [13]	0.3 [13]
Malleus	1.9 × 10^3^ [34]	1.41 × 10^10^ [35]	0.3 [13]
Incus	1.9 × 10^3^ [34]	1.41 × 10^10^ [35]	0.3 [13]
Stapes	1.9 × 10^3^ [34]	1.41 × 10^10^ [35]	0.3 [13]
Tympanic annulus	1.2 × 10^3^ (assumed)	6 × 10^5^ [13]	0.3 [13]
Manubrium	1.0 × 10^3^ [12]	4.7 × 10^9^ [12]	0.3 [13]
Tensor tympanic tendon	2.5 × 10^3^ [12]	2.6 × 10^6^ [12]	0.3 [13]
Lateral malleolar ligament	2.5 × 10^3^ [12]	6.7 × 10^4^ [12]	0.3 [13]
Anterior malleolar ligament	2.5 × 10^3^ [12]	2.1 × 10^6^ [12]	0.3 [13]
Superior malleolar ligament	2.5 × 10^3^ [12]	4.9 × 10^4^ [12]	0.3 [13]
Posterior incudal ligament	2.5 × 10^3^ [12]	6.5 × 10^6^ [14]	0.3 [13]
Stapedial tendon	2.5 × 10^3^ [12]	5.2 × 10^5^ [12]	0.3 [13]
Stapedial annular ligament	2.5 × 10^3^ [12]	2 × 10^5^ [15]	0.3 [13]
Incudomalleolar joint	3.2 × 10^3^ [13]	1.41 × 10^10^ [13]	0.3 [13]
Incudostapedial joint	1.2 × 10^3^ [13]	6 × 10^5^ [36]	0.3 [13]

**Table 2:** Modal frequencies for the tympanic-ossicular system (TOS) FE model.

Mode number	Frequency (Hz)	Main pattern	Mode number	Frequency (Hz)	Main pattern	Mode number	Frequency (Hz)	Main pattern
1	266.9	LT	11	880.55	J	21	1865	LT
2	267.03	LT	12	970.76	TM	22	1866	LT
3	330.82	J, LT	13	1073.3	TM	23	1894	LT
4	562.27	LT	14	1552.7	TM	24	2032	TM
5	580.91	LT	15	1725.5	LT	25	2343	TM
6	612.84	LT	16	1750.8	LT	26	2527	TM
7	613.67	LT	17	1751.2	LT	27	2572	J
8	704.62	TM	18	1782.7	LT	28	2683	TM, J
9	853.5	LT	19	1809.4	TM	29	2683	TM, J
10	854.34	LT	20	1831	LT	30	2765	TM

## Data Availability

The data used to support the findings of this study are available from the corresponding author upon request.
